# Voice pitch is negatively associated with sociosexual behavior in males but not in females

**DOI:** 10.3389/fpsyg.2023.1200065

**Published:** 2023-07-11

**Authors:** Alvaro Mailhos, Damián-Amaro Egea-Caparrós, Álvaro Cabana, Francisco Martínez-Sánchez

**Affiliations:** ^1^Facultad de Psicología, Universidad de la República, Montevideo, Uruguay; ^2^Facultad de Psicología, Universidad de Murcia, Murcia, Spain

**Keywords:** voice pitch, fundamental frequency, sociosexuality, sociosexual behavior, sexual selection

## Abstract

Acoustic cues play a major role in social interactions in many animal species. In addition to the semantic contents of human speech, voice attributes – e.g., voice pitch, formant position, formant dispersion, etc. – have been proposed to provide critical information for the assessment of potential rivals and mates. However, prior studies exploring the association of acoustic attributes with reproductive success, or some of its proxies, have produced mixed results. Here, we investigate whether the mean fundamental frequency (*F0*), formant position (*Pf*), and formant dispersion (*Df*) – dimorphic attributes of the human voice – are related to sociosexuality, as measured by the Revised Sociosexual Orientation Inventory (*SOI-R*) – a trait also known to exhibit sex differences – in a sample of native Spanish-speaking students (101 males, 147 females). Analyses showed a significant negative correlation between *F0* and sociosexual behavior, and between *Pf* and sociosexual desire in males but not in females. These correlations remained significant after correcting for false discovery rate (*FDR*) and controlling for age, a potential confounding variable. Our results are consistent with a role of *F0* and *Pf* serving as cues in the mating domain in males but not in females. Alternatively, the association of voice attributes and sociosexual orientation might stem from the parallel effect of male sex hormones both on the male brain and the anatomical structures involved in voice production.

## Introduction

Insect stridulations, amphibian choruses, bird songs, and mammalian vocalizations – among other acoustic signals and cues – play a major role in a broad range of animal taxa. In addition to communicating, e.g., the presence of a predator, or the position and distance to a food source, animal acoustic cues and signals broadcast a wealth of information about the caller, which allows the receiver to assess the potential mate value of an individual of the opposite sex or weigh the risk posed by a prospective rival (see Bradbury and Vehrencamp, 2011).

In primates, vocalizations are produced by the vibration of the vocal folds as air passes through and the resulting pulses are further modified by the structure of supralaryngeal elements (Taylor et al., 2016). In human adults, the larynx and supralaryngeal structures are sexually dimorphic (Lieberman, 1986; Taylor et al., 2016) and the resulting acoustic signals reflect such sex differences. Typically, adult male and female voices differ in mean fundamental frequency (e.g., Bachorowski and Owren, 1999; Feinberg et al., 2018; Aung and Puts, 2020) – the mean vibration frequency of the vocal folds (Ghazanfar and Rendall, 2008) – and formant frequencies (e.g., Fant, 1966; Puts et al., 2012; Cartei et al., 2014) – the resonances of the vibrating air in the vocal tract during speech production (Ghazanfar and Rendall, 2008). While the former is mainly determined by the length and mass of the vocal folds, the latter are determined by the length and cross-sectional area of the vocal tract (Lieberman, 1986; Ghazanfar and Rendall, 2008). Although male and female voices also differ in other acoustic attributes, studies addressing voice dimorphism have usually focused on *F0* and related variables, and formant frequencies or derived indices, e.g., formant dispersion (*Df*) – the average distance between successive formants (Fitch, 1997) – and formant position (*Pf*) – the average standardized formant value for the first – usually four – formants (Puts et al., 2012).

Male voice pitch – the perceptual correlate of *F0* (Ghazanfar and Rendall, 2008) – is affected by sex hormones throughout development. In this direction, for instance, male voice pitch was found to correlate with self- and third-party-rated masculinity (Pereira et al., 2019). During puberty, an increase in testosterone levels drives the thickening of the vocal cords, which in turn results in a significant drop in voice pitch (Evans et al., 2008); similar effects are also observed during testosterone therapy in female-to-male gender transitioning (Irwig et al., 2016). In addition to the effects on the vocal cords, testosterone has important anabolic and ergogenic properties (Handelsman et al., 2018); because of this, several studies have explored the association of dimorphic voice attributes with strength and/or physical dominance. Previous studies found a negative association of male *F0* (Hodges-Simeon et al., 2014; Mailhos et al., 2022), *Pf* (Puts et al., 2012), and the standard deviation of the fundamental frequency (Puts et al., 2012) with physical strength. Other studies, however, failed to find a link between these variables (Sell et al., 2010; Atkinson et al., 2012; Han et al., 2018).

Although in modern societies interpersonal conflicts are rarely settled by fighting or physical strength, physical formidability has been linked to perceived dominance and authority. It has been proposed that physical formidability might have become associated with social status, leadership, and dominance over the course of human evolution (Murray, 2014; Lukaszewski et al., 2016). In this regard, Ko et al. (2015) observed that participants who were assigned to a more powerful role in a negotiation game showed a lower and more monotone pitch than participants assigned to a less powerful role. Similarly, in group settings, it has been observed that raising one’s pitch early during a negotiation predicted a low rank, while a decrease in voice pitch was associated with a high emerging rank (Cheng et al., 2016). In line with these results, Leongómez et al. (2017) observed that males and females who were low in self-perceived dominance increased their pitch more in the presence of a dominant listener than when interacting with a neutral one.

A growing body of evidence suggests that voice attributes are also related to perceived trustworthiness and fidelity. Low-pitched voices have been associated with perceived trustworthiness both in males and females (Tsantani et al., 2016; Schild et al., 2019). However, while Schild and colleagues also observed a significant negative association between mean fundamental frequency and reported infidelity, they did not find a link between voice attributes and trustworthy behavior in economic games. Furthermore, in a recent meta-analysis, these authors found that a lower mean *F0* – i.e., voice pitch – might be indeed a valid cue for self-reported infidelity (Schild et al., 2021).

Other studies have explored the association between voice attributes and reproductive success, or some of its proxies, in humans. Apicella et al. (2007) observed a negative association between fundamental frequency and actual reproductive success, i.e., the number of children born to Hadza men – but not to women – even after controlling for age. Similarly, Puts et al. (2006) found a trend where male participants with lower *F0* reported a higher number of sexual partners – this trend, however, was found not to be statistically significant. In mammals, including the human species, males can greatly increase their reproductive success by inseminating a large number of females (Buss, 1998), hence the current or desired number of sexual partners or sociosexuality orientation measurements have been used as proxies of reproductive success in males. In the same vein, Hughes et al. (2004) found that opposite-sex ratings of voice attractiveness were negatively correlated with the age of first sexual intercourse, and the reported number of sex partners and extra-pair copulations both in males and females. In turn, Hill et al. (2013) showed that vocal masculinity – a compound measure of voice pitch and formant dispersion – interacts with physical dominance to significantly predict mating success in males. Furthermore, a recent meta-analysis that explored the association of voice pitch and the mating domain – mating attitudes and behavior – in males showed a significant association of those variables (*r* = 0.132 [0.061,0.204], *q* = 0.0012; Lidborg et al., 2022). Notwithstanding the former results, other studies reported an association between voice attributes and the reproductive domain in females, but not in males. Atkinson et al. (2012), for instance, found that *F0* was a predictor of different reproductive success indicators, including the number of children and grandchildren, in Himba females, but not males.

The groundbreaking work of Kinsey et al. (1948, 1953) allowed for the identification of individual differences in sociosexual behavior and attitudes, including frequency of actual and preferred sexual intercourse, number of actual and preferred sexual partners, and tendency to engage in uncommitted sexual relationships. Simpson and Gangestad (1991) developed the Sociosexual Orientation Inventory (*SOI*), one of the most widely used instruments to measure this internal dimension or tendency towards unrestricted sociosexual behavior. Sex differences in sociosexuality are generally large and have been observed cross-culturally, although such differences might be modulated environmentally (Schmitt, 2005). That is, sex differences seem to be larger in demanding reproductive environments, whereas sex differences in sociosexuality are more moderate in countries with more political and economic gender equality. Penke and Asendorpf (2008) proposed a revised version of the original one-dimensional *SOI*– the Revised Sociosexual Orientation Inventory (*SOI-R*) – and showed that sociosexuality consists of three facets: sociosexual behavior, attitude, and desire, rather than a single dimension. Interestingly, Valentova et al. (2019) observed that male speech *F0* was negatively associated with sociosexuality in males but not in females; the relationship between male singing *F0* and sociosexuality was, however, inverted.

This study was aimed, thus, at furthering our understanding of the role of human voice cues in mating behavior. To achieve this, we explored the association of several dimorphic attributes of the human voice – i.e., mean fundamental frequency, formant position, and formant dispersion – with the different facets of sociosexual orientation, i.e., sociosexual behavior, attitude, and desire, as measured by *SOI-R* (Penke and Asendorpf, 2008).

## Materials and methods

### Voice recordings and acoustic analyses

Male and female students at Universidad de Murcia were invited to participate in a study aimed at exploring the association between sociosexual behavior and voice attributes. One hundred one self-reported heterosexual male and 147 female students (male median age = 20.52 years, interquartile range [*IQR*] = 2.34 years; female median age = 20.28 years, *IQR* = 2.20 years) from a mid-sized Spanish university participated in this study in exchange for course credit. As to ensure participants’ privacy and favor the honesty of the answers, students completed all questionnaires in separate private cubicles. All questionnaires and voice recordings were identified with a numerical code, and only the researcher team had access to the data. The procedure was approved by the Local Research Ethics Board, and all participants gave written informed consent.

Participants provided voice recordings by reading the following text: “El trabajo parece interesante y además el salario es muy bueno” (The job seems interesting and the salary is also very good). All participants were native speakers of Spanish. Recordings were made with an AKG D3700S cardioid microphone and a Fostex FR-2LE recorder at a sampling rate of 44,100 Hz and 16-bit quantization. All recordings were made by the same researcher keeping all conditions unchanged in a quiet, non-soundproofed room, placing the microphone 8 cm away from the speaker’s mouth at an angle of 45 degrees to avoid aerodynamic noise. Recordings were saved as uncompressed wav files. Data collection took place in 2019, prior to the outbreak of COVID-19 in Spain. The tasks took participants about 20–30 min to complete.

Voice recordings were analyzed using the software package Praat version 6.0.25 (Boersma and Weenink, 2017). The fundamental frequency was calculated following the software developers’ recommendations; fundamental frequencies F1 through F4 were calculated as described in Valentova et al. (2019), while *Df*, *Pf*, were calculated as described by Fitch (1997), Reby and McComb (2003), and Puts et al. (2012), respectively. The calculation of *Pf* involves scaling the formant values using z-scores. We calculated the mean and standard deviation of formant values using a bootstrapping procedure to balance the sample with respect to gender. For each of 1.000 replicates, 124 males and 124 females were sampled with reposition from the original dataset, resulting in a balanced sample with respect to gender, with the same number of total observations. For each replicate, the mean and standard deviation of the formant values was computed. We then used the mean of these values across all the replicates to compute the z-scores in the original dataset.

### Sociosexuality measurement

All participants completed also the paper-and-pencil version of the Revised Sociosexual Orientation Inventory (*SOI-R*) (Penke, 2011). *SOI-R* is a 9-item self-report questionnaire aimed at assessing interindividual differences in the tendency to engage in sexual relationships without deeper emotional commitment. This instrument allows for the assessment of three facets of sociosexuality: behavior (past behavioral experiences), attitude (attitude towards uncommitted sex), and desire (heightened sexual interest towards potential partners without romantic commitment). In our sample, the instrument showed acceptable internal consistency, with a Cronbach’s alpha value of 0.84, similar to the value reported in the original study (Penke, 2011).

### Statistical analyses

Two women and four men failed to provide their birth date or provided an inconsistent date of birth (e.g., date of data collection), similarly, one man skipped one item in the SOIR1 facet of the SOIR scale. The pairwise deletion method was used to deal with missing data points in the correlation analyses (see below).

All data analyses were conducted with R software (v4.1.0; R Core Team, 2021). Shapiro–Wilk tests of normality indicated that with the exception of *Pf*, all variables were non-normally distributed.

Associations between sociosexual variables and acoustic attributes were thus explored by means of Spearman rank correlation analysis. All tests were two-tailed, and the significance level was set to *α* = 0.05. In order to avoid false positive findings, the Benjamini and Hochberg false discovery rate (*FDR*; Benjamini and Hochberg, 1995) was used to correct multiple comparisons with *FDR* < 0.05 considered as statistically significant. Bootstrapping provides an alternative to relying on underpowered samples. Thus, the significance of the correlations was also assessed through this method with 10,000 simulation iterations.

## Results

Descriptive statistics of the speakers’ sociosexual and acoustic variables used in this study are shown in Table 1. A Mann–Whitney test showed significant differences in mean fundamental frequency, formant position, and formant dispersion between male and female voices. Similarly, males and females differed in all three dimensions of sociosexuality (SOIR1: sociosexual behavior; SOIR2: sociosexual attitude; and SOIR3: sociosexual desire).

**Table 1:** Descriptive statistics for sociosexual and acoustic variables and Mann–Whitney test results.

	Males			Females			Mann–Whitney test	
	*Mean*	*Median*	*IQR*	*Mean*	*Median*	*IQR*	*U*	Value of *p*
*F0*	118.77	118.61	18.59	207.92	206.67	24.86	0	<0.001
*Pf*	−1.11	−1.12	0.89	1.12	1.12	1.03	171	<0.001
*Df*	1057.32	1058.35	67.35	1184.10	1185.87	81.75	1,079	<0.001
*SOIR1*	7.16	6.00	4.00	5.82	4.00	2.5	8807.5	<0.01
*SOIR2*	19.51	20.00	9.00	17.58	18.00	9.5	8959.5	<0.01
*SOIR3*	15.27	16.00	10.00	9.64	8.00	7.0	11133.5	<0.001
Age	20.77	20.52	2.34	20.62	20.28	2.2	7414.5	0.475

*F0*, mean fundamental frequency; *Pf*, formant position; *Df*, formant dispersion; *SOIR1*, sociosexual behavior; *SOIR2*, sociosexual attitude; *SOIR3*, sociosexual desire.

Because acoustic and sociosexual variables were not normally distributed, in order to analyze the relationship between these variables, Spearman rank correlation coefficients were calculated. Tables 2, 3 show the correlations between sociosexual and acoustic variables for males and females, respectively.

**Table 2:** Zero-order Spearman correlations of males’ sociosexual variables and acoustic attributes.

	*F0*	*Pf*	*Df*	*SOIR1*	*SOIR2*	*SOIR3*
*F0*						
*Pf*	0.02 *n* = 101 *p* = 1.000					
*Df*	−0.08 *n* = 101 *p* = 0.791	0.51 *n* = 101 *p* = 0.000				
*SOIR1*	−0.27 *n* = 100 *p* = 0.023	−0.22 *n* = 100 *p* = 0.076	−0.05 *n* = 100 *p* = 0.970			
*SOIR2*	−0.13 *n* = 101 *p* = 0.427	−0.17 *n* = 101 *p* = 0.252	−0.15 *n* = 101 *p* = 0.283	0.43 *n* = 100 *p* = 0.000		
*SOIR3*	−0.09 *n* = 101 *p* = 0.713	−0.33 *n* = 101 *p* = 0.003	−0.06 *n* = 101 *p* = 0.947	0.10 *n* = 100 *p* = 0.683	0.34 *n* = 101 *p* = 0.002	
Age	0.02 *n* = 97 *p* = 1.000	−0.16 *n* = 97 *p* = 0.283	−0.05 *n* = 97 *p* = 0.992	0.20 *n* = 97 *p* = 0.137	0.02 *n* = 97 *p* = 1.000	−0.09 *n* = 97 *p* = 0.713

*F0*, mean, mean fundamental frequency; *Pf*, formant position; *Df*, formant dispersion, *SOIR1*, sociosexual behavior; *SOIR2*, sociosexual attitude, and *SOIR3*, sociosexual desire. Value of *p*s are corrected for false discovery rate (*FDR*).

**Table 3:** Zero-order Spearman correlations of females’ sociosexual variables and acoustic attributes.

	*F0*	*Pf*	*Df*	*SOIR1*	*SOIR2*	*SOIR3*
*F0*						
*Pf*	0.16 n = 147 *p* = 0.103					
*Df*	0.01 *n* = 147 *p* = 1.000	0.68 *n* = 147 *p* = 0.000				
*SOIR1*	−0.17 *n* = 147 *p* = 0.103	−0.07 *n* = 147 *p* = 0.621	−0.03 *n* = 147 *p* = 0.918			
*SOIR2*	−0.15 *n* = 147 *p* = 0.136	−0.19 *n* = 147 *p* = 0.054	−0.20 *n* = 147 *p* = 0.042	0.34 n = 147 p = 0.000		
*SOIR3*	−0.10 *n* = 147 *p* = 0.396	−0.17 *n* = 147 *p* = 0.103	−0.09 *n* = 147 *p* = 0.468	0.22 *n* = 147 *p* = 0.019	0.39 *n* = 147 *p* = 0.000	
Age	−0.08 *n* = 145 *p* = 0.484	−0.10 *n* = 145 *p* = 0.396	−0.21 *n* = 145 *p* = 0.028	0.38 *n* = 145 *p* = 0.000	0.14 *n* = 145 *p* = 0.209	−0.02 *n* = 145 *p* = 0.988

*F0*, mean fundamental frequency; *Pf*, formant position; *Df*, formant dispersion; *SOIR1*, sociosexual behavior; *SOIR2*, sociosexual attitude; and *SOIR3*, sociosexual desire. Value of *p*s are corrected for false discovery rate (*FDR*).

Mean fundamental frequency (*F0*) showed a significant negative correlation with sociosexual behavior (SOIR1) in males but not in females, and formant position exhibited a significant negative correlation with sociosexual desire (SOIR3).

The confidence intervals of all correlations were calculated by means of bootstrapping with 10,000 iterations. All significant correlations were supported by this analysis (see Supplementary Tables S1, S2). In addition, the correlation between male *Pf* and sociosexual behavior (SOIR1) that approached significance by Spearman rank correlation analysis (*r*_100_ = −0.22, *p* = 0.076), reached statistical significance by estimation of the confidence intervals for this correlation (95% *CI* [−0.406, −0.025]).

Because age showed significant correlations with both *Df* and sociosexual behavior in females, partial Spearman rank correlations were calculated. After controlling for age, only the correlation between *F0* and sociosexual behavior in males, and the correlation between *Pf* and sociosexual desire in males remain significant (see Table 4).

**Table 4:** Partial Spearman correlations of sociosexual variables and voice acoustic attributes controlling for age.

	*F0*	*Pf*	*Df*
Males			
*SOIR1*	−0.31*	−0.19	−0.04
*SOIR2*	−0.14	−0.16	−0.16
*SOIR3*	−0.09	−0.33**	−0.04
Females			
*SOIR1*	−0.14	−0.03	0.06
*SOIR2*	−0.14	−0.18	−0.18
*SOIR3*	−0.1	−0.16	−0.09

*F0*, mean fundamental frequency; *Pf*, formant position; *Df*, formant dispersion; *SOIR1*, sociosexual behavior; *SOIR2*, sociosexual attitude; *SOIR3*, sociosexual desire. *p < 0.05; **p < 0.01.

## Discussion

Voice pitch, the perceptual correlate of mean *F0*, is one of the most salient features of the human voice and it is also one of the attributes that show the highest degree of sexual dimorphism. Prior studies point to a role of voice pitch in both intrasexual competition and intersexual selection processes. Our results show a negative correlation between mean fundamental frequency and sociosexual behavior – as measured by the behavior facet of the Revised Sociosexual Orientation Inventory (Penke and Asendorpf, 2008) – in males (*r_100_* = −0.27, *p* = 0.023; 95%CI = [−0.45, −0.07]) but not in females (*r_147_* = −0.17_,_ *p* = 0.103, *95%CI* = [−0.32, −0.00]); in other words, our findings show a trend where men with lower-pitched voices exhibit a more unrestricted sexual behavior. This association is in line with previous studies which have shown that male-speech lower *F0* is related to higher scores on the *SOI-R* scale (Valentova et al., 2019; Stern et al., 2021) and that males with lower-pitched voices have higher reproductive success and more children born to them (Apicella et al., 2007). While Stern et al. (2021) did find an association between voice pitch and sociosexuality in women, neither the studies by Apicella et al. (2007) and Valentova et al. (2019) nor the present study found an association between female *F0* and sociosexuality.

As mentioned before, *F0* and other male voice attributes have been found to provide relevant cues to several biologically relevant attributes of the speaker (e.g., height, physical strength, dominance, trustworthiness, but also self-reported infidelity). Given that male and female reproductive strategies differ – while male reproductive strategies seem to be quantity-oriented, female strategies appear to be quality-oriented – male cues play an important role in reproductive behavior informing receptive females about the mate value of their potential mates.

Sociosexual behavior has been proposed to reflect an individual’s overall allocation of effort (e.g., time, energy, or money) to short-term versus long-term mating strategies (Penke and Asendorpf, 2008). Thus, the correlation between *F0* and sociosexual behavior might reflect this internal disposition of the male speakers. Alternatively, because sociosexual behavior (facet 1 of *SOI-R*) is a composite score that takes into account the number of sexual partners in the last 12 months, lifetime number of single-occasion sexual partners, and number of sexual encounters without an interest in a long-term committed relationship (Penke and Asendorpf, 2008), this variable may as well be affected by the potential partners’ disposition to have sex with the respondent. In this scenario, the correlation between *F0* and sociosexual behavior might be in line with previous studies which have shown a consistent female preference for lower-pitched male voices (for a review, see: Pisanski and Feinberg, 2018). Such a preference might be the result of the negative association of voice pitch with body size and physical formidability (Pisanski et al., 2014; Aung and Puts, 2020) which are generally thought to be honest indicators of a male’s ability to pass genes that will increase the survival or reproductive success of her offspring.

The Spearman rank correlation analysis between *Pf* and sociosexual behavior (SOIR1) and correlation confidence intervals study for this correlation produced mixed results. That is, while the first failed to reach significance, the confidence intervals analysis seems to support a significant correlation between the variables. Further studies will be needed to confirm or reject the true association of the variables. Once again, since *Pf* has been associated with desirable male attributes, this feature of the male voice may contribute to broadcasting the genetic quality or physiological state of the male to potential mates; or this might simply result from the joint action of male sexual hormones on the brain – thus promoting more typical male behaviors – and peripheric structures.

This study also shows that *Pf* is associated with sociosexual desire – a motivational state characterized by sexual interest often linked to subjective sexual arousal and sexual fantasies (Penke and Asendorpf, 2008). While the association between voice pitch and sociosexual behavior in males may reflect an adaptive role of *F0* in signaling fighting ability or biological quality to potential rivals or mates – that is, males with lower *F0* may have a higher number of sexual partners by deterring romantic rivals or by attracting romantic partners – it is more difficult to think of an adaptive function of broadcasting sexual desire. We think the latter correlation probably relies on the simple fact that vocal cords and the neural structures involved in sexual motivation in males are under the effect of the same androgenizing hormones.

Some shortcomings of the present study should be mentioned. The current study analyzes only some of the most widely used acoustic variables. We cannot rule out the possibility that other variables, on their own, or via complex interaction patterns may reflect more accurately an individual’s sociosexuality. In addition, the sample used in this study consisted exclusively of university students; in the future, a more representative sample should be included. Also, while it poses some methodological problems, we believe the use of free speech in a dating or seduction context would much better reflect a person’s sociosexual behavior.

Finally, future research may focus on testing these results in different language groups, and exploring whether the reported association of sociosexual behavior and desire with acoustic features of the male voice are perceptually relevant.

To conclude, in this study, we identified a significant association between *F0* and sociosexual behavior, and between *Pf* and sociosexual desire in males but not in females. While we cannot rule out the possibility that these associations reflect the joint effects of male sex hormones on both the brain and peripheral anatomic structures, our findings are consistent with a role of male voice attributes in advertising desirable genetic and/or physiological attributes for potential mates. By analyzing the relationship between acoustic features of the human voice and sociosexual behavior in a sample of native Spanish-speaking men and women, this study contributes to furthering our understanding of the role of the human voice in the reproductive domain.

## Data availability statement

The raw data supporting the conclusions of this article will be made available by the authors, without undue reservation.

## Ethics statement

The studies involving human participants were reviewed and approved by Comité de Ética de Investigación, Facultad de Psicología, Universidad de Murcia. The patients/participants provided their written informed consent to participate in this study.

## Author contributions

AM and FM-S proposed and designed the study. AM, FM-S, and D-AE-C collected the data. AM and ÁC conducted the data analysis. AM, FM-S, and ÁC wrote the manuscript. All authors contributed to the article and approved the submitted version.

## Conflict of interest

The authors declare that the research was conducted in the absence of any commercial or financial relationships that could be construed as a potential conflict of interest.

## Publisher’s note

All claims expressed in this article are solely those of the authors and do not necessarily represent those of their affiliated organizations, or those of the publisher, the editors and the reviewers. Any product that may be evaluated in this article, or claim that may be made by its manufacturer, is not guaranteed or endorsed by the publisher.
