# Supplementary material for: Voice pitch is negatively associated with sociosexual behavior in males but not in females
Source: Front Psychol. 2023 Jul 11;14:1200065. doi: 10.3389/fpsyg.2023.1200065 (PMC10367086; doi:10.3389/fpsyg.2023.1200065)
Supplement: Supplementary file 1 [file Data_Sheet_1.docx]

**SUPPLEMENTARY MATERIALS**

***Supplementary Materials Table 1.*** *Confidence intervals of Spearman correlations of males’ sociosexual variables and acoustic attributes.*

| Correlation | 95% CI | |
| --- | --- | --- |
|  | Lower limit | Upper limit |
| *F0* & *Pf* | -0.206 | 0.244 |
| *F0* & *Df* | -0.270 | 0.125 |
| *F0* & *SOIR1* | -0.453 | -0.072 |
| *F0* & *SOIR2* | -0.324 | 0.070 |
| *F0* & *SOIR3* | -0.281 | 0.113 |
| *F0* & Age | -0.200 | 0.230 |
| *Pf* & *F0* | -0.206 | 0.244 |
| *Pf* & *Df* | 0.403 | 0.664 |
| *Pf* & *SOIR1* | -0.406 | -0.025 |
| *Pf* & *SOIR2* | -0.360 | 0.026 |
| *Pf* & *SOIR3* | -0.503 | -0.136 |
| *Pf* & Age | -0.342 | 0.059 |
| *Df* & *F0* | -0.270 | 0.125 |
| *Df* & *Pf* | 0.403 | 0.664 |
| *Df* & *SOIR1* | -0.258 | 0.151 |
| *Df* & *SOIR2* | -0.347 | 0.052 |
| *Df* & *SOIR3* | -0.253 | 0.133 |
| *Df* & Age | -0.257 | 0.158 |
| *SOIR1* & *F0* | -0.453 | -0.072 |
| *SOIR1* & *Pf* | -0.406 | -0.025 |
| *SOIR1* & *Df* | -0.258 | 0.151 |
| *SOIR1* & *SOIR2* | 0.269 | 0.577 |
| *SOIR1* & *SOIR3* | -0.084 | 0.280 |
| *SOIR1* & Age | 0.010 | 0.380 |
| *SOIR2* & *F0* | -0.324 | 0.070 |
| *SOIR2* & *Pf* | -0.360 | 0.026 |
| *SOIR2* & *Df* | -0.347 | 0.052 |
| *SOIR2* & *SOIR1* | 0.269 | 0.577 |
| *SOIR2* & *SOIR3* | 0.140 | 0.522 |
| *SOIR2* & Age | -0.195 | 0.237 |
| *SOIR3* & *F0* | -0.281 | 0.113 |
| *SOIR3* & *Pf* | -0.503 | -0.136 |
| *SOIR3* & *Df* | -0.253 | 0.133 |
| *SOIR3* & *SOIR1* | -0.084 | 0.280 |
| *SOIR3* & *SOIR2* | 0.140 | 0.522 |
| *SOIR3* & Age | -0.317 | 0.133 |
| Age & *F0* | -0.200 | 0.230 |
| Age & *Pf* | -0.342 | 0.059 |
| Age & *Df* | -0.257 | 0.158 |
| Age & *SOIR1* | 0.010 | 0.380 |
| Age & *SOIR2* | -0.195 | 0.237 |
| Age & *SOIR3* | -0.317 | 0.133 |
| Note. *F0*: mean fundamental frequency, *Pf*: formant position, *Df*: formant dispersion, *SOIR1*: sociosexual behavior, *SOIR2*: sociosexual attitude, *SOIR3*: sociosexual desire. | | |

***Supplementary Materials Table 2.*** *Confidence intervals of Spearman correlations of females’ sociosexual variables and acoustic attributes.*

| Correlation | 95% CI | |
| --- | --- | --- |
|  | Lower limit | Upper limit |
| *F0* & *Pf* | -0.005 | 0.328 |
| *F0* & *Df* | -0.159 | 0.173 |
| *F0* & *SOIR1* | -0.321 | 0.000 |
| *F0* & *SOIR2* | -0.316 | 0.011 |
| *F0* & *SOIR3* | -0.272 | 0.074 |
| *F0* & Age | -0.239 | 0.073 |
| *Pf* & *F0* | -0.005 | 0.328 |
| *Pf* & *Df* | 0.556 | 0.758 |
| *Pf* & *SOIR1* | -0.222 | 0.094 |
| *Pf* & *SOIR2* | -0.346 | -0.020 |
| *Pf* & *SOIR3* | -0.305 | -0.010 |
| *Pf* & Age | -0.256 | 0.078 |
| *Df* & *F0* | -0.159 | 0.173 |
| *Df* & *Pf* | 0.556 | 0.758 |
| *Df* & *SOIR1* | -0.194 | 0.138 |
| *Df* & *SOIR2* | -0.348 | -0.041 |
| *Df* & *SOIR3* | -0.239 | 0.070 |
| *Df* & Age | -0.377 | -0.039 |
| *SOIR1* & *F0* | -0.321 | 0.000 |
| *SOIR1* & *Pf* | -0.222 | 0.094 |
| *SOIR1* & *Df* | -0.194 | 0.138 |
| *SOIR1* & *SOIR2* | 0.188 | 0.490 |
| *SOIR1* & *SOIR3* | 0.049 | 0.392 |
| *SOIR1* & Age | 0.234 | 0.521 |
| *SOIR2* & *F0* | -0.316 | 0.011 |
| *SOIR2* & *Pf* | -0.346 | -0.020 |
| *SOIR2* & *Df* | -0.348 | -0.041 |
| *SOIR2* & *SOIR1* | 0.188 | 0.490 |
| *SOIR2* & *SOIR3* | 0.240 | 0.531 |
| *SOIR2* & Age | -0.036 | 0.304 |
| *SOIR3* & *F0* | -0.272 | 0.074 |
| *SOIR3* & *Pf* | -0.305 | -0.010 |
| *SOIR3* & *Df* | -0.239 | 0.070 |
| *SOIR3* & *SOIR1* | 0.049 | 0.392 |
| *SOIR3* & *SOIR2* | 0.240 | 0.531 |
| *SOIR3* & Age | -0.191 | 0.144 |
| Age & *F0* | -0.239 | 0.073 |
| Age & *Pf* | -0.256 | 0.078 |
| Age & *Df* | -0.377 | -0.039 |
| Age & *SOIR1* | 0.234 | 0.521 |
| Age & *SOIR2* | -0.036 | 0.304 |
| Age & *SOIR3* | -0.191 | 0.144 |
| Note. *F0*: mean fundamental frequency, *Pf*: formant position, *Df*: formant dispersion, *SOIR1*: sociosexual behavior, *SOIR2*: sociosexual attitude, *SOIR3*: sociosexual desire. | | |
